# Effect of Maternal or Formulated Transition Milk on the Health and Performance of Dairy Calves

**DOI:** 10.3390/ani13101674

**Published:** 2023-05-17

**Authors:** Ana Paula da Silva, Amanda M. Cezar, Ariany F. Toledo, Sophia C. Dondé, Marina G. Coelho, Cristiane R. Tomaluski, Gercino F. Virgínio Júnior, Carla M. M. Bittar

**Affiliations:** 1 Department of Animal Science, “Luiz de Queiroz” College of Agriculture, University of São Paulo, Av. Pádua Dias, n 11, São Paulo 1341-900, Brazil; annaps@usp.br (A.P.d.S.); amandamcezar@usp.br (A.M.C.); arianytoledo@usp.br (A.F.T.); cattleya.donde@gmail.com (S.C.D.); marinacoelho.vet@gmail.com (M.G.C.); cristiane.tomaluski@usp.br (C.R.T.); 2 Minas Gerais Agricultural Research Agency, Experimental Field of Montes Claros, Montes Claros 39404-128, Brazil; gercino.junior@epamig.br

**Keywords:** colostrum powder, early nutrition, newborn calf

## Abstract

**Simple Summary:**

Decreases in morbidity rate and increases in performance are essential to obtain replacement heifers in a reasonably productive and cost-efficient manner. The aim of this study was to understand the value of feeding maternal or formulated transition milk to newborn Holstein calves after colostrum feeding for three days. After receiving high-quality colostrum in one meal, during the following six meals calves were fed 2 L of either whole milk, transition milk or formulated transition milk, by the addition of colostrum powder to whole milk. After that, calves were fed equally, receiving 6 L/d of whole milk, and starter and water were free choice. Even though calves fed transition milk or formulated transition milk presented a higher total solids intake, there were no effects on health or performance. The transition milk composition and the number of meals after colostrum feeding need more investigation.

**Abstract:**

The study aimed to evaluate the effect of maternal or formulated transition milk with colostrum powder on the performance and health of dairy calves. After receiving 12% of their birth weight in high-quality colostrum, 36 Holstein calves (17 males and 19 females) were blocked according to sex, birth date and birth weight (29.16 kg ± 1.34) and randomly distributed into three treatments: (1) Milk: supplying 4 L/d of whole milk (WM) for three days (*n* = 12); (2) Transition milk (TM): supplying 4 L/d of maternal transition milk for three days (*n* = 12); (3) Formulated transition milk (FTM): supplying 4 L/d of whole milk enriched with 280 g/d of colostrum powder, for three days (*n* = 12). Daily feeding was split into two feedings, and after the sixth feeding of the transition diet calves were fed 6 L/d of whole milk and had ad libitum access to water and calf starter until 56 d, when the study ended. Calves fed TM or FTM presented a higher total solids intake (*p* < 0.05). Concentrations of glucose (*p* = 0.096) and lactate (*p* = 0.063), evaluated from 0 h to 72 h, tended to be higher in WM-fed calves compared to TM. There were no effects on calf’s health or performance and weight; at the week 8 averaged 65.06 kg ± 1.85. All treatments resulted in adequate performance and good health, however, the potential benefits of providing TM or FTM were not seen in this study. The transition milk composition and the number of meals after colostrum feeding need more investigation.

## 1. Introduction

Infectious disease resulting in morbidity and mortality has a significant impact on economic losses within calf rearing [1]. Thus, management practices that reduce the occurrence of diseases, especially in the first weeks of the calf’s life, are essential to the efficiency of rearing systems.

After the colostrum feeding period, the supply of transition milk has been widely discussed as a feed management strategy that can minimize disease risks and increase performance in rearing heifers [2]. There is some evidence that performance improvement occurs because transition milk contains large amounts of biologically active nutritive and non-nutritive substances that benefit intestinal development [3,4]. In addition, NASEM [5] recommends feeding transition milk during the second and third days of life to improve calf health and performance.

Transition milk can be defined as the secretion produced by the mammary gland in the intermediate period between the production of colostrum and whole milk. According to Godden [6], after the first milking (defined as colostrum), the period between the second and sixth postpartum milking is considered transition milk, as there is a gradual decline in the content of nutritional and bioactive components at each milking.

In addition to an extremely vulnerable digestive system, the newborn calf needs an extra energy supply to adjust to the extrauterine life. Nutritionally, transition milk contains higher concentrations of fat, protein, amino acids and bioactive compounds than whole milk [7]. All of these compounds may have nutritional, physiological and metabolic benefits for the newborn calf.

Feeding transition milk promotes extended IgG consumption, benefiting the calves’ health. Although the immunoglobulins in transition milk do not result in greater IgG absorption due to the intestinal closure process [6], IgG can reduce infections caused by enteric viruses and bacteria. This reduction occurs by the formation of a layer that prevents the attachment of microorganisms to the intestinal wall, providing local protection [8]. Thus, the abrupt change from colostrum to milk replacer (MR) or whole milk (WM) excludes an important nutritional transition phase, in which cows still produce nutrient- and energy-rich secretions, hormones and bioactive compounds important for animal development [9]. The main bioactive compounds in colostrum and bovine transition milk are immunoglobulins, insulin-like growth factors (IGF-I and IGF-II), insulin, lactoferrin, lysozyme and lactoperoxidase [7].

Since providing transition milk is important, colostrum powder can supplement WM or MR in the first days of life to mimic the composition of maternal transition milk [3]. In addition, this possibility of mimicking transition milk can make it an alternative to providing a more nutritious diet with low microbial contamination, rich in bioactive compounds, and with a standardized supply of IgG for producers for whom maternal transition milk is unavailable, or those who do not want to affect milking parlor logistics and routine. Van Soest et al. [3] recommend that transition milk formulated with colostrum powder should have approximately 15 g/L of IgG per meal to improve weight gain and health.

Based on this information, this research hypothesizes that providing a treatment diet with higher levels of IgG and bioactive compounds in the first 3 days of life improves the performance and health of dairy calves. Thus, the present study aims to evaluate how the supply of maternal transition milk or transition milk formulated with colostrum powder affects the performance and health of dairy calves.

## 2. Materials and Methods

### 2.1. Experimental Site and Animals

The study was conducted from February to July 2021 at the Animal Science Department of the Luiz de Queiroz College of Agriculture—ESALQ/USP, located in Piracicaba-SP. Thirty-six Holstein calves (male *n* = 17 and female *n* = 19) from the university dairy herd were enrolled in the study. The experimental design was a randomized block, with animals allocated in blocks according to date of birth, birth weight and sex.

After birth, the calves were immediately separated from their dams, weighed on a mechanical scale (ICS-300, Coimma Ltd.a, Dracena, Brazil) and had their navels disinfected with 7% iodine solution. Colostrum feeding was provided by supplying high-quality colostrum (23% to 25% Brix) from the dam or the colostrum bank (not pooled) within the first 3 h of life in a volume corresponding to 12% of birth weight. Colostrum was supplied through a bottle or esophageal tube when necessary.

The calves remained housed in individual suspended pens (113 × 140 cm) until they were 14 days old and were then tethered in wood hutches in an open field until they were 56 days old, when the study ended. The stalls were cleaned daily by replacing the sawdust bed, and the shelters were moved to avoid the accumulation of feces and to keep the calves clean and dry. Hutches were placed in an east–west lateral orientation to ensure access to shade.

### 2.2. Experimental Groups

In the next feeding after colostrum feeding, which occurred in the afternoon (5 pm) for calves born in the morning and in the morning (7 am) for those born on the afternoon of the previous day, calves were fed one of three transition diets for three days, after which they started to receive whole milk:

(1) Whole Milk (WM): supplying 4 L/d of whole milk, divided into two meals;
(2) Transition milk (TM): supplying 4 L/d of maternal transition milk, divided into two meals;
(3) Formulated transition milk (FTM): supplying 4 L/d of whole milk enriched with 70 g/L of colostrum powder (or 15 g IgG/L; Alta Genetics, Brazil, SCCL^®^, Saskatoon, SK, Canada), divided into two meals.

The transition feeding period from colostrum to the whole milk lasted three days, during which time all calves received six meals containing their respective treatments by bottle. The TM was standardized to 16% Brix, the second to fourth milking of the cows from the University herd having been collected for 3 months before the trial started and frozen in 2 L meals. The 16% Brix value corresponds to approximately 30 g/L of IgG, considering mean values observed in the third and fourth milking after calving [10]. Transition milk was thawed and heated to 38 °C before feeding it to the calves.

The colostrum and transition milk samples fed to each calf were collected in sterilized plastic tubes with a volume of 20 mL and stored in a freezer (−20 °C) for later analysis of standard plate count (SPC) as described below.

### 2.3. Microbiological Quality of Colostrum and Transition Milk

A sample of colostrum, which each animal received, and three samples of transition milk (one per day) corresponding to the three days of supply of the treatment were collected, totaling 36 samples of colostrum and 108 samples of transition milk. Transition milk samples were composed by calf, so there were 12 samples per treatment for the analysis. The microbial quality was evaluated in duplicates using the standard plate count methodology [11]. Briefly, serial dilution was performed with distilled water, using an automatic pipette with adjustable volume (Eppendorf^®^ Research, São Paulo, Brazil) and disposable sterile tips. The dilution was 1 mL of colostrum in 9 mL of distilled water, followed by serial dilution up to 10^−6^. For inoculation, 1 mL of colostrum diluted to 10^−6^ was pipetted and poured into the center of a dedicated Total Bacterial Count Plate (CompactDry^®^ TC, Nissui Pharmaceutical Co. Ltd., Tokyo, Japan). Subsequently, the plates were incubated for 48 h in a BOD oven (TE-391, Technal Equipamentos Científicos, Piracicaba, Brazil) at 37 ± 1 °C. Results were read through the CFU Visual Count; for this purpose, a photo was taken of each plate on a flat surface, with a white background and good lighting for subsequent counting of the colonies. Each plate was counted three times by two different counters, and the average of the counts was used as the final CFU value. To calculate the total plate count (TPC) values for each plate, the following equation was used, which considers a sample dilution rate of 10^−6^:

TPC (thousand CFU/mL) = (n° CFU × 1,000,000)/1000.

### 2.4. Blood Parameters

Blood samples were taken on 4 time points; at birth prior to the first colostrum feed (0 h) and during the 3 days transition period from colostrum to treatment diet (24 h, 48 h and 72 h), at least 2 h after feeding. The samples were collected through jugular vein puncture, using three different evacuated tubes (VACUETTE do Brazil, Campinas, SP, Brazil) containing: (1) sodium fluoride as an antiglycolytic and potassium EDTA as an anticoagulant to obtain plasma; (2) potassium EDTA, to evaluate the hematocrit, erythrogram and leukogram, as described later; and (3) a clot activator, to obtain serum. Hematocrit was determined in a capillary using a microhematocrit centrifuge, Model SPIN 1000 (MICROSPIN). After collection, the samples were quickly transported to the laboratory and centrifuged. tubes 1 and 3 were centrifuged at 2000× *g* for 20 min, at a temperature of 4 °C to obtain plasma or serum, and stored in the freezer at −20 °C for further analysis. The blood parameters were determined using an Automatic System for Biochemistry—Model SBA—200 (CELM, Barueri, SP, Brazil). LABTEST Diagnóstica S.A. (Lagoa Santa, MG, Brazil) kits were used for glucose, total protein and lactate determinations. A kit from RANDOX Laboratories—Life Sciences Ltd. (Crumlin, UK) was used to determine non-esterified fatty acids (NEFA).

An aliquot of serum from tube 3, collected 48 h after colostrum supply, was used to evaluate the transfer of passive immunity, using the Brix refractometer and, in addition, through the IgG concentration using a commercial ELISA kit (Bovine IgG ELISA Kit, catalog nº E11-118; Bethyl Laboratories Inc., Montgomery, TX, USA). The apparent efficiency absorption (AEA) was calculated [12] using the following equation:

AEA IgG (g) = {[serum IgG g/L 24 h − serum IgG g/L birth] × birth weight kg × 0.09}/IgG intake (g), where: 0.09 = plasma volume (9% of BW).

After the transition period from colostrum to a treatment diet, blood samples were collected weekly, always 2 h after the morning milk feed, to trace the biochemical and metabolic profiles following the same methodology performed in the first 72 h of life.

### 2.5. Performance and Health in the Preweaning Period

After the 6th meal of the transition liquid diet feeding, calves received 6 L/d of whole milk divided into two meals (7:00 a.m. and 5:00 p.m.) through nipple-buckets, and any refusals were recorded. Whole milk was collected every 2 days in the milking parlor in plastic milk cans and refrigerated at 5 °C. Milk samples were taken weekly from the milk can just prior to heat and then feed to the calves. They were analyzed for fat, protein and lactose by Fourier transform infrared spectroscopy [13] and for SCC by flow cytometry (Clínica do Leite, Piracicaba, Brazil—Table 1). The total solids consumption of TM and FTM was calculated, considering 195.6 g/L of total solids for the FTM (70 g/L of powdered colostrum added with 12.56% of whole milk solids) and 180 g/L of total solids for the TM (16% Brix) [14].

The calves had ad libitum access to water and starter concentrate (22.6% CP, 3.5% EE, 4.5% Ash, 31.4% NDF, 20.9% ADF, 38.0% NFC; Nutrimax, Salto de Pirapora, SP, Brazil) from the second day of life. The concentrate was supplied daily in the morning, and leftovers were weighed on a digital scale (Toledo 9094, São Bernardo do Campo, SP, Brazil) to calculate the daily calf starter intake. The pre-weaning period comprised eight weeks, when the experimental period ended. After the end of the study calves were gradually weaned, but this period was not considered in the present data.

Calves were weighed at birth and weekly until the eighth week of age, always before the morning feeding. Average daily gain (ADG) and feed efficiency (kg of BW gain/kg of total DMI) were calculated for the pre-weaning period (0–56 d). Body measurements were taken every 14 d. The wither height and the hip width were measured using a ruler with a scale in centimeters, and the heart girth was measured using a flexible tape with a scale in centimeters.

Health was monitored daily until the end of experiment, and all occurrences of diseases and treatments were recorded in an individual file. The occurrence of diarrhea was monitored through the fecal score, as follows: (0) normal, (1) pasty or semi-formed; (2) fluid; (3) liquid-aqueous [15]. Calves were considered to have diarrhea when a fecal score ≥2 was recorded for two consecutive days, at which point they were provided with oral rehydration treatment 4 h after milk feeding, consisting of 5 g of NaCl, 25 g of dextrose and 10 g of bicarbonate for each liter. Antibiotics were administered per the veterinarian’s recommendations when the animal presented, in addition to diarrhea, symptoms such as fever (rectal temperature ≥39.4 °C) and refusal to ingest a treatment diet or whole milk feed. Clinical signs such as a cough, nasal and ocular discharge and fever were considered for respiratory diseases, according to the Calf Health Scoring Criteria developed by the University of Wisconsin–Madison. For bovine tick fever, calves were evaluated for anemia by visualization of eye and mouth mucosa, as well as by hematocrit values (<25%) determined in the same way described above.

### 2.6. Statistical Analysis

Performance measures (concentrate intake, ADG, heart girth, wither height and hip width), fecal scores, erythrocyte count, white blood cell count and blood parameters (glucose, total protein and NEFA) were analyzed as repeated measures over time using the MIXED procedure of the SAS statistical package (version 9.4, SAS Institute Inc., Cary, NC, USA), according to the model: Y_ijk_ = μ + T_i_ + B_j_ + e_ij_ + W_k_ + TW_ik_ + e_ijk_. Where, Y_ijk_ = response variable; μ = overall mean; T_i_ = treatment effect (different protocols of transition milk or colostrum); B_j_ = block effect; e_ij_ = residual error A; W_k_ = effect of animal age (hours or days of life); TW_ik_ = effect of treatment and age interaction; e_ijk_ = residual error B.

The covariance matrices, “compound symmetry, heterogeneous compound symmetry, autoregressive, heterogeneous autoregressive, unstructured, banded, variance components, Toeplitz, antidependence and heterogeneous Toeplitz”, were tested and defined according to the lowest value obtained for Akaike’s Information Criterion corrected (AICC). The model included the effects of treatment, week (age of calves) and the interaction between treatment and week as fixed effects. The block effect was included in the model as a random effect. The subject of the repeated measures was the animal undergoing treatment.

Passive immunity transfer variables (serum Brix, serum IgG and AEA) and health variables (days with fever, days with diarrhea and the number of treatments for diarrhea, respiratory illnesses and BTF) were analyzed as non-repeating variables using the following statistical model: Y_ji_ = μ + D_i_ + B_j_ + e_ij_. Where, Y_ji_ = response variable; μ = overall mean; D_i_ = effect of transition milk or colostrum feeding protocols; B_j_ = block effect; e_ij_ = residual error. The method of least squares (LSMEANS) was used to compare means with significance *p* < 0.05 and trends 0.05 < *p* < 0.10.

## 3. Results

### 3.1. Transition Milk Total Plate Count

The average TCP of colostrum was 143,968 CFU/mL, while the average TCP for the WM, TM and FTM groups were 102,167 CFU/mL, 136,150 CFU/mL and 113,083 CFU/mL, respectively (Table 2).

### 3.2. Transfer of Passive Immunity and Transition Milk Intake

Immunoglobulin intake was not different for the three treatment groups, and calves had successful passive transfer with no differences among the groups and values above 9.4% Brix (Table 3). Likewise, there was no difference among the experimental groups for the IgG concentration, determined through the ELISA test, as well as the apparent efficiency of absorption. In addition, the volume of the transition diet consumed did not differ between treatments. However, it was lower than outlined (12 L) due to the animals’ refusal to ingest. However, the TM and FTM groups presented a higher consumption of total solids during the transition from colostrum to a treatment diet than the WM-fed group (*p* = 0.042).

### 3.3. Blood Parameters of 0 h and 48 h of Life

The concentrations of albumin, total protein and NEFA evaluated at birth (0 h) and during the period that transition diet was offered (24 h, 48 h and 72 h) were not influenced by the diet in the transition period (Table 4). However, there was a trend for glucose (*p* = 0.096) and lactate concentrations (*p* = 0.063) to be higher for calves fed WM than those fed TM, with no differences for FTM treatment. As expected, all blood parameters showed a time effect (*p* < 0.001; Supplementary Figure S1). Albumin and total protein concentrations increased from 0 h to 72 h. Glucose concentration values also increased up to 48 h but decreased at 72 h. Conversely, the lactate and NEFA concentration decreased from birth to 72 h of life.

### 3.4. Health

**Figure 1:**
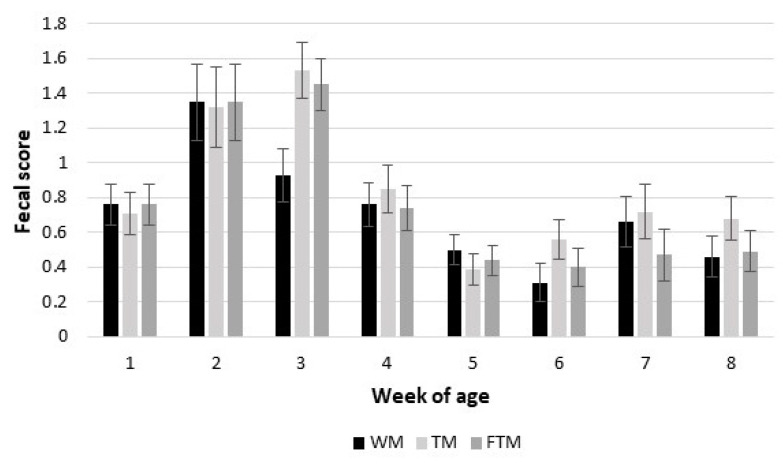
Mean fecal score according to the age, by week, of calves fed whole milk, transition milk or formulated transition milk for three days after colostrum feeding. WM = whole milk; TM = transition milk; FTM = formulated transition milk. *p* < 0.394 for treatment effect, *p* < 0.001 for age effect and *p* < 0.429 for the treatment and age interaction effect.

The treatment diet did not have an effect on the mean fecal score (Table 5), days with diarrhea, days with fever or the number of antibiotic treatments for diarrhea (*p* > 0.05). A diarrhea episode was considered when the fecal score was ≥2. The highest frequency of diarrhea occurred during the second and third weeks of life for all groups (Figure 1). After this period, the mean fecal score remained below one, indicating a low incidence of diarrhea during the experimental period (56 d), regardless of the treatment diet.

Treatments did not influence other health variables such as hematocrit, number of treatments for respiratory diseases and bovine tick fever (*p* > 0.05). In general, all groups had a low incidence of health problems.

### 3.5. Intake and Performance in the Preweaning Period

Total dry matter intake and starter intake were not affected by the treatment diet. Starter intake increased as calves aged, however, there were no differences between treatments throughout the experimental period, or at the eighth week of life (Table 6). In addition, the average body weight, average daily gain (ADG), feed efficiency, average hip width and heart girth and gains/week withers-height were not influenced by the diet in the transition period. All intake variables, performance and body measurements increased over the weeks (*p* < 0.05; Supplementary Figure S2). However, coinciding with the higher occurrence of diarrhea, the ADG was reduced from an average of 650 g/d at the first week to about 500 g/d during the second and third weeks of age (Supplementary Figure S2).

### 3.6. Blood Parameters during Pre-Weaning

Treatments did not affect albumin, total protein, glucose and lactate concentrations (*p* > 0.05). However, all blood parameters showed an age effect (*p* < 0.05; Table 7; Supplementary Figure S3). Albumin concentration showed little variation during the evaluated weeks, with only a slight increase in the eighth week of age. The total protein concentration showed erratic behavior during the evaluated weeks, showing a decline from weeks two (9.75 mg/dL) to five (7.80 mg/dL), with a concentration peak at the sixth week (9.9 mg/dL). Glucose concentration varied slightly and lactate concentration decreased during the evaluated period.

## 4. Discussion

According to the suggestions of Lombard et al. [16] for the management of passive transfer, all groups were classified in the excellent category, with an IgG concentration higher than 25 g/L and Brix values above 9.4%. Calves with IgG levels ≥25 g/L at 48 h of age have lower morbidity, a higher survival rate and are less likely to die during preweaning period [16,17]. Even though calves received only one colostrum feeding of 12% of their birth weight, immunoglobulin intake was high at approximately 510 g on average, an amount 70% higher than recommended to minimize morbidity and mortality rates in herds (300 g of IgG) in two colostrum meals [16,17]. Our colostrum management resulted in high serum IgG levels, thus resulting in a low occurrence of health problems and improved performance, considering the preweaning nutrition program. However, this management may have reduced the opportunity to show significant effects for transition milk feeding after colostrum feeding.

Another point that may have reduced the chance of showing the benefits of providing TM or FTM in the present study was the volume and period of transition diets provided. Treatments were designed to provide 4 L/d, divided into two meals (morning and afternoon) for three days, totaling an intake of 12 L during the transition. However, calves ingested an average of 11 L of their respective treatments from the second to the fourth day of life, and one could argue that this may be insufficient to express short-term benefits. Van Soest et al. [3] showed superiority in ADG and reduced overall inflammation in calves that received maternal TM and the milk replacer supplemented by colostrum powder, compared to animals fed only the milk replacer. They provided 17.1 L divided into nine meals. Calves from the present study ingested about 37.7% of their birth weight as transition milk during the first three days of age, while calves from Van Soest et al. [3] ingested about 45% of their birth weight during the first three days of age, but divided into three meals per day. The number of meals after colostrum feeding and the transition milk composition to benefit neonatal calves regarding health and performance need further investigation.

NASEM [5] recommends providing transition milk during the second and third days of life to improve the health and performance of calves. However, the volume and number of days of transition milk feeding are variable in the literature [2,3,18,19]. Van Soest et al. [3] supplied three transition milk meals/d in a volume of 1.89 L from the second to the fourth day of life. Kargar et al. [2] replaced non-marketable milk with transition milk at four inclusion levels (0 L, 0.5 L, 1 L and 2 L) over the 21 d period. Conneely et al. [19] evaluated the supply of two or four additional meals of transition milk in a volume of 4 L, with each meal compared to the control treatment without transition milk. Steinhoff-Wagner et al. [18] evaluated the supply of milkings three and five on the second and third day of life, respectively, in a volume corresponding to 10% of the BW. Our decision to feed calves during 3 d or six meals, with a TM with an average of 16% Brix, was based on data showing how long or for how many milkings cows produce TM [7].

Another widely discussed factor is the mass of immunoglobulins and bioactive compounds that should be contained in the transition milk for the benefits to be apparent. Van Soest et al. [3] provided 10 g/kg of IgG DM through maternal transition milk, or 98 g/kg of IgG DM through the milk replacer supplemented by colostrum powder. However, pioneering studies evaluating transition milk supply did not assess the concentrations of IgG provided [18,19]. Whether it is preferable to feed more meals with a lower IgG and bioactive compounds content or feed a lower number of meals with higher IgG and bioactive compounds content is still debated. The volume, number of meals, the mass of immunoglobulins or % Brix and the content of bioactive compounds of the transition milk to be fed require further studies.

As expected, the serum protein concentration was low for all groups after birth. After the ingestion of colostrum, there was a marked increase in total protein concentrations for all groups due to the absorption of colostral immunoglobulins. This increase was expected since there is a positive correlation between total serum protein values and serum concentration of immunoglobulins in the first days of the calf’s life [20]. Although the supply of TM or FTM provides prolonged intake of IgG [4], which could influence total protein values, the absorption of immunoglobulins through the intestinal epithelium decreases linearly with time from birth to about 24 h of life as a response of the gut closure [21]. Thus, similar total protein concentrations between treatments were expected. However, the presence of immunoglobulins in the intestine 24 h after birth provides local protection by forming a layer that prevents the attachment of microorganisms to the intestinal wall [8]. The bioactive compounds in the transition milk or the mixture of milk or milk replacer with colostrum powder can promote early intestinal development and provide local protection, resulting in greater weight gain [3,22].

Glucose concentrations, evaluated during the transition from colostrum to milk, tended to be higher for calves fed WM than those fed TM and FTM. This trend may have occurred because TM contains lower lactose concentrations and higher fat and casein concentrations than whole milk [4]. Inabu et al. [23], in contrast to the results of the present study, found no treatment effect for glucose concentration in calves receiving whole milk, a mixture of 50% colostrum and 50% milk, simulating transition milk or colostrum for up to 72 h after the birth. However, as in the present study, plasma glucose concentrations increased with the evaluated time. Steinhoff-Wagner et al. [18] evaluated the provision of the third and fifth milking after calving on the second and third days of life, respectively, or a milk-based formula with a nutrient content similar to transition milk. The authors concluded that plasma glucose concentrations were higher for calves that received transition milk when compared to calves that received a milk-based formula. These results indicate that the supply of transition milk is important for maintaining blood glucose in the first days of life, which is not observed when formulas are fed.

Lactate is one of the newborn calves’ most important precursors for gluconeogenesis [24]. Regardless of the transition diet, plasma lactate concentrations decreased after birth up to 72 h of life, however, calves fed with WM tended to have higher lactate concentrations than those fed with TM. These results suggest that calves fed WM had greater metabolization of energy reserves, showing that providing TM and FTM can benefit the maintenance of energy homeostasis in newborn calves. In addition to differences in nutrient composition, FTM and TM allow for greater consumption of solids in the first days of feeding life.

Although NEFA concentrations did not show differences among transition diets, it behaved similarly to lactate concentrations, reducing their concentration from birth to 72 h of life. Van Soest et al. [4], evaluating the supply of TM or milk replacer in the first 4 days of life, also found no effects on NEFA concentrations. Based on the behavior of the glucose, lactate and NEFA metabolites, all diets showed adequate energy supply, although it was expected that TM and FTM would be superior due to their composition having a higher concentration of solids, protein and fat when compared to the composition of WM [7]. Unfortunately, in the present study, it was not possible to evaluate the composition of TM and FTM. We can only speculate that they provided a higher concentration of solids since the second and third milking were used to compose the TM, and for the FTM the amount of 70 g/L of colostrum powder was added to milk.

Health variables were not affected by treatments, possibly due to the excellent passive transfer achieved by all calves, which may have decreased the chance of demonstrating the potential benefits of transition milk supply. As discussed earlier, the number of meals, the IgG and bioactive content may have also contributed to this. However, as the number of calves used for this study was calculated with a focus on understanding the effects on performance, this may have limited the opportunity to understand the effects on morbidity. Nevertheless, Van Soest et al. [3], evaluating 35 calves per treatment, also did not detect differences in health parameters when evaluating the supply of milk replacer, transition milk or milk replacer supplemented with colostrum powder for three days after colostrum feeding, probably because of the adequate colostrum feeding protocol with an intake of 300 g IgG. However, animals fed with 350 mL of colostrum +4.65 L of whole milk or with 700 mL of colostrum +4.3 L of whole milk for 14 days had a reduction in days with diarrhea when compared to animals that received exclusively 5 L of milk in the same period [25]. This reduction may indicate that a prolonged supply of TM may be more efficient regarding health variables than a short-period supply in the first days of life. Prolonged colostrum feeding also benefits calves’ intestinal development [22], health [26] and performance [25]. However, an effect of providing TM and FTM on health variables was expected, since both treatment diets have high concentrations of IgG and other bioactive compounds, which have antimicrobial and anti-inflammatory properties.

Although no differences were observed for the number of days with diarrhea, TM-fed calves had an average of three more days with a fecal score ≥2. None of the evaluated treatment diets underwent the pasteurization process, but the TM treatment had higher TPC (CFU/mL), meaning that this treatment had a higher microbial load than WM and FTM, which may have influenced the higher average of days with fecal score ≥2. Studies show that when pasteurization is performed properly, it can reduce the microbial load of colostrum and bulk tank milk [27,28], thereby reducing the occurrence of infectious diseases such as diarrhea. In a study conducted by Armengol [28], calves fed colostrum and pasteurized milk had a lower percentage of morbidity (5.2%) compared to calves fed colostrum and unpasteurized milk (15%) during the first 21 days of age. Based on this, we speculate that the high microbial load present in TM and FTM may also have contributed to the lack of effects of providing transition milk on the performance and health of calves. Proper handling of transition milk, whether maternal or formulated, its storage and the time of its supply are essential for maintaining a low TPC to guarantee a high-quality diet for young calves. Feeding formulated transition milk may be a good alternative to reducing transition liquid diet contamination, if the SCC of the milk used is low. That would increase the odds of having beneficial effects of feeding a transition diet to calves, from colostrum to whole milk, with regard to performance and health.

However, it is also noted that the three groups had a few days with fever, indicating a low occurrence of severe inflammatory and infectious processes, probably due to the high consumption of IgG in colostrum. In addition, the results suggest a low sanitary challenge in the experimental period, since all treatments had low morbidity and no mortality, which may also have contributed to the reason why the benefits of providing TM or FTM were absent. According to Van Soest et al. [3], the low morbidity and mortality rate during the experimental period made it difficult to detect any differences in health parameters when providing milk replacer, transition milk or milk replacer supplemented with colostrum powder for three days after the colostrum.

It is important to note that the supply of FTM resulted in satisfactory values for health and performance variables, and promoted a higher consumption of solids than in the animals that received milk. Thus, this can be an alternative to providing a more nutritious diet rich in bioactive compounds in the first days of life for producers for whom maternal transition milk is unavailable, or those who do not want to affect the logistics and routine of the milking parlor. In addition, it is an alternative that guarantees the provision of a transition diet with low microbial contamination due to management.

The intake and performance variables did not differ according to the treatment diet after colostrum. As opposed to what was expected, calves fed WM presented higher withers height than calves fed FTM, which cannot be explained by the other performance data. The results of the present study differ from the literature, which has shown higher rates of gain for calves fed with transition milk or formulated transition milk [2,3]. In a similar study, Van Soest et al. [3] showed that the provision of maternal transition milk and formulated transition milk resulted in higher ADG (0.616 and 0.620 kg/d, respectively) than the milk replacer group (0.562 kg/d). In the present study, the ADG was higher than those observed by Van Soest et al. [3]. However, it is important to highlight that the present research used whole milk during the pre-weaning period and not a milk replacer, as in Van Soest et al. [3].

The biochemical parameters evaluated weekly showed values within the reference range for all groups. There was a decrease in total protein concentrations at weeks three, four and five, with a normal decline due to the reduction of colostrum antibodies in circulation. This occurs because maternal immunoglobulins have an average half-life of 30 days [29], and the calf has not yet started to produce enough antibodies to compensate for the serum losses of total protein. This decline in total protein values also coincides with a higher frequency of diarrhea (third week), in which the calves are more susceptible to infections.

The plasma glucose concentrations found in the present study are values above those reported in the literature for calves fed whole milk and of similar age [30]. During the pre-weaning phase lactate values decreased with advancing age, which may be associated with the establishment of liver function in the first weeks, with lactate being converted into glucose via gluconeogenesis, which may also have contributed to maintaining glucose concentrations elevated.

## 5. Conclusions

All feeding practices after colostrum resulted in adequate performance considering the nutrition program and good health conditions. However, the potential benefits of providing TM or FTM were not observed, probably due to the low volume ingested and the short period of supply of transition diets (3 d). More research is needed to understand the best feeding period and composition of transition milk to benefit the calf’s health and performance.

## Figures and Tables

**Table 1:** Chemical composition of whole milk fed to calves after the different transition liquid diets.

	Whole Milk ^1^	SD
Total Solids, %	12.56	1.12
Fat, %	3.85	1.19
Protein, %	3.34	0.10
Lactose, %	4.41	0.236
Urea Nitrogen, mg/dL	13.43	1.569
Casein, %	2.59	0.09
SCC ^2^, ×1000 cells/mL	636	390

^1^ Whole milk collected weekly (*n* = 12) from the milk can before feeding to calves; ^2^ somatic cell count by flow cytometry.

**Table 2:** Total plate count (CFU/mL) of colostrum and the three different transition liquid diets: whole milk, transition milk and formulated transition milk.

		Total Plate Count (CFU/mL)			
Feed ^1^	*n*	Average	Minimum	Maximum	SD
Colostrum	36	143,968	57,000	262,883	53,682.1
WM	12	102,167	17,000	241,000	60,833.3
TM	12	192,409	51,667	755,000	195,107.2
FTM	12	113,083	19,000	352,000	106,684.3

^1^ WM = whole milk; TM = transition milk; FTM = formulated transition milk.

**Table 3:** Serum Brix, TM volume intake from calves fed whole milk, transition milk or formulated transition milk.

Item	Treatments ^1^			SEM	*p*-Value
	WM	TM	FTM		
Ig, Intake g ^2^	509.9	495.5	522.4	23.25	0.365
Brix, % 48 h	9.8	10.6	10.1	0.29	0.103
IgG, g/L 48 h	49.0	56.0	44.2	4.07	0.139
AEA ^3^, %	25.1	28.6	23.1	3.02	0.194
Volume of TM Intake, L	11.22	11.26	11.02	0.258	0.775
Total Solids of Treatment Diet ^4^, kg	1.41 ^b^	2.02 ^a^	2.15 ^a^	0.042	0.001

^a,b^ Means within a row with different superscripts are significantly different (*p* ≤ 0.05); ^1^ WM = whole milk; TM = transition milk; FTM = formulated transition milk; ^2^ IgG intake considering only colostrum supply; ^3^ apparent efficiency absorption; ^4^ intake of total solids of treatment diet during 3 d of life.

**Table 4:** Blood parameters collected at 0, 24, 48 and 72 h of life of calves fed whole milk, transition milk or formulated transition milk for 3 days after colostrum feeding.

Item	Treatments ^1^			SEM	*p*-Value ^2^		
	WM	TM	FTM		T	H	TxH
Albumin, g/dL	2.98	2.91	2.86	0.074	0.439	0.001	0.444
Total Protein, g/dL	8.64	9.45	8.81	0.385	0.286	0.001	0.457
Glucose, mg/dL	122.99 ^a^	110.38 ^b^	108.86 ^ab^	4.974	0.096	0.001	0.386
Lactate, mg/dL	32.09 ^a^	25.64 ^b^	26.92 ^ab^	1.973	0.063	0.001	0.954
NEFA ^3^, mmol/L	0.44	0.38	0.34	0.044	0.286	0.001	0.258

^a,b^ Means within a row with different superscripts are significantly different (*p* ≤ 0.05); ^1^ WM = whole milk; TM = transition milk; FTM = formulated transition milk; ^2^ T = treatment effect; H = effect of the evaluation hour; T × H = effect of interaction between treatment and evaluation hour; ^3^ NEFA = non-esterified fatty acids.

**Table 5:** Fecal score and health of calves fed whole milk, transition milk or formulated transition milk for 3 days after colostrum feeding.

Item	Treatments ^1^			SEM	*p*-Value ^2^		
	WM	TM	FTM		T	A	TxA
Fecal Score	0.7	0.8	0.7	0.06	0.394	0.001	0.429
Hematocrit, %	27.21	28.56	26.89	0.917	0.239	0.001	0.713
Fever Days	2.83	1.90	3.74	0.630	0.143	-	-
Days with Diarrhea	7.72	10.48	9.72	1.482	0.378	-	-
Number of Treatments							
Antibiotics	0.41	0.45	0.91	0.246	0.285	-	-
Diarrhea	0.33	0.45	0.41	0.181	0.891	-	-
Respiratory Disease	0.08	0.0	0.08	0.068	0.611	-	-
Bovine Tick Fever	0.08	0.08	0.08	0.083	1.000	-	-

^1^ WM = whole milk; TM = transition milk; FTM = formulated transition milk; ^2^ T = treatment effect; A = age effect; T × A = effect of interaction between treatment and age of evaluation.

**Table 6:** Intake, performance and body measurements of calves fed whole milk, transition milk or formulated transition milk for 3 days after colostrum feeding.

Item	Treatments ^1^			SEM	*p*-Value ^2^		
	WM	TM	FTM		T	A	T × A
Intake, g DM/d							
Total	949.1	937.2	949.3	25.09	0.927	0.001	0.557
Concentrate	208.3	203.4	209.1	23.17	0.980	0.001	0.964
Concentrate at 8th wk	576.9	562.8	552.6	95.95	0.956	-	-
BW, Kg							
Birth	29.0	28.2	30.3	1.34	0.158	-	-
At the 8th wk	65.8	63.6	65.8	1.85	0.493	-	-
ADG, g/day	667.8	616.8	634.5	19.94	0.178	0.001	0.361
Gain to Feed Ratio	0.705	0.663	0.683	0.023	0.307	0.001	0.276
Body Measures, cm							
Heart Girth	83.94	82.38	83.17	0.774	0.3724	<0.0001	0.8399
Hip Width	21.06	20.77	20.92	0.158	0.4322	<0.0001	0.3777
Withers Height	82.92	81.57	82.33	0.446	0.5473	<0.0001	0.1024

^1^ WM = whole milk; TM = transition milk; FTM = formulated transition milk; ^2^ T= treatment effect; A= age effect; T × A= effect of interaction between treatment and age.

**Table 7:** Preweaning blood parameters of calves fed whole milk, transition milk or formulated transition milk for 3 days after colostrum feeding.

Item	Treatments ^1^			SEM	*p*-Value ^2^		
	WM	TM	FTM		T	A	T × A
Glucose, mg/dL	143.8	148.7	138.3	3.95	0.130	0.001	0.723
Lactate, mg/dL	14.0	14.5	14.6	0.539	0.660	0.001	0.780
Total Protein, g/dL	8.3	8.6	8.6	0.23	0.593	0.001	0.249
Albumin, g/dL	3.8	3.4	3.4	0.038	0.750	0.001	0.472

^1^ WM = whole milk; TM = transition milk; FTM = formulated transition milk; ^2^ T = treatment effect; A = age effect; T × A = effect of interaction between treatment and age

## Data Availability

The data presented in this study are available on request from the corresponding author. The data are not publicly available due to restrictions by the research group.

## Supplementary Materials

The following supporting information can be downloaded at: https://www.mdpi.com/article/10.3390/ani13101674/s1, Figure S1. Blood parameters collected at 0, 24, 48 and 72 h of life of calves fed whole milk, transition milk or formulated transition milk for 3 days after colostrum feeding. *p*-value for treatment or treatment and age interaction effect are all >0.05. *p*-value for age effect are all <0.0001; Figure S2. Starter intake, average daily gain and body measurements of calves fed whole milk, transition milk or formulated transition milk for 3 days after colostrum feeding. *p*-value for treatment or treatment and age interaction effect are all >0.05. *p*-value for age effect are all <0.0001; Figure S3. Preweaning blood parameters of calves fed whole milk, transition milk or formulated transition milk for 3 days after colostrum feeding.
