# Supplementary material for: Effect of Maternal or Formulated Transition Milk on the Health and Performance of Dairy Calves
Source: Animals (Basel). 2023 May 17;13(10):1674. doi: 10.3390/ani13101674 (PMC10215583; doi:10.3390/ani13101674)
Supplement: Supplementary file 1 [file animals-13-01674-s001.zip › animals-2390787-supplementary.pdf]

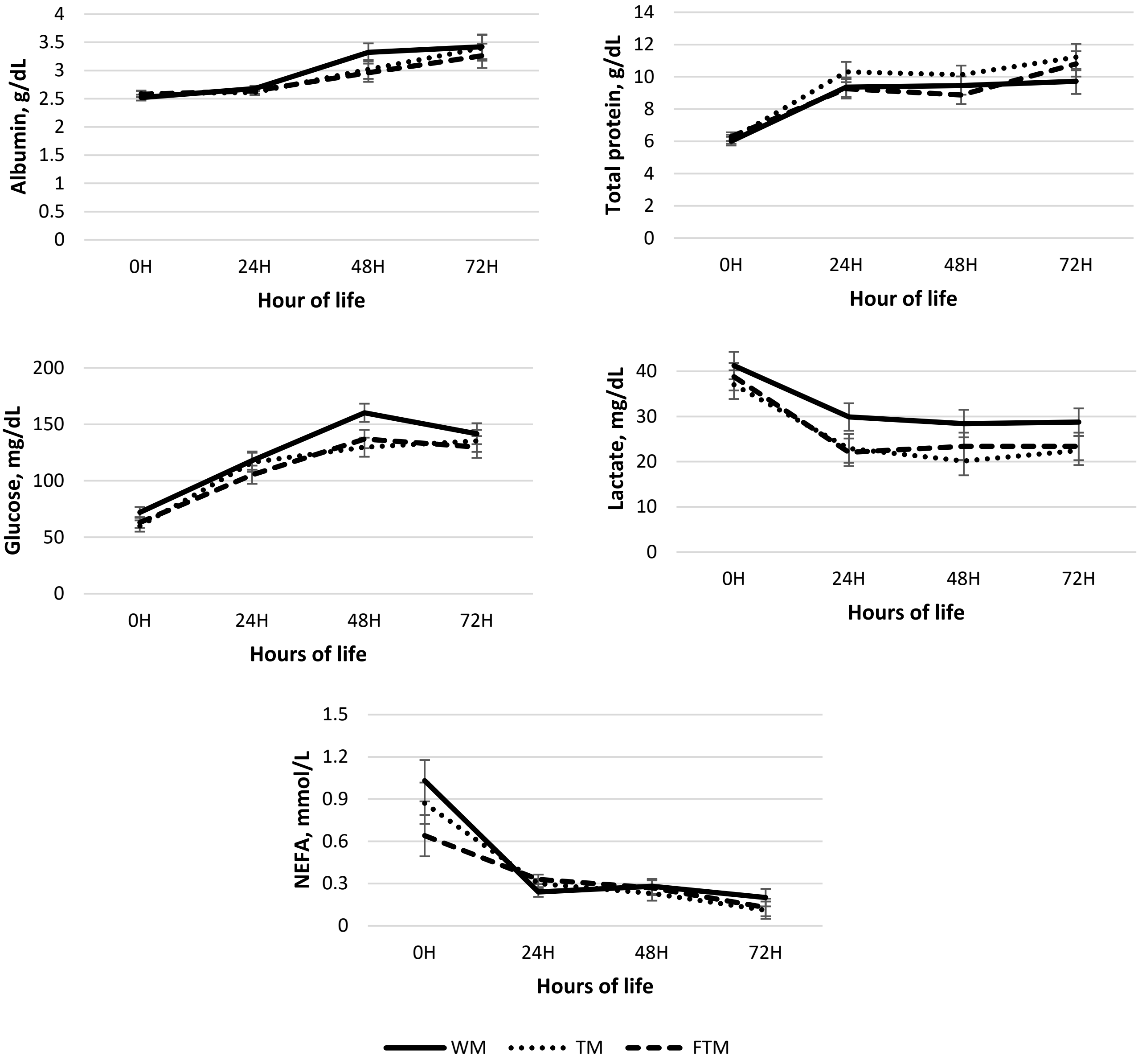

Figure S1. Blood parameters collected at 0, 24, 48 and 72h of life of calves fed whole milk, transition milk or formulated transition milk for 3 days after colostrum feeding. P-value for treatment or treatment and age interaction effect are all > 0.05. P-value for age effect are all <0.0001.

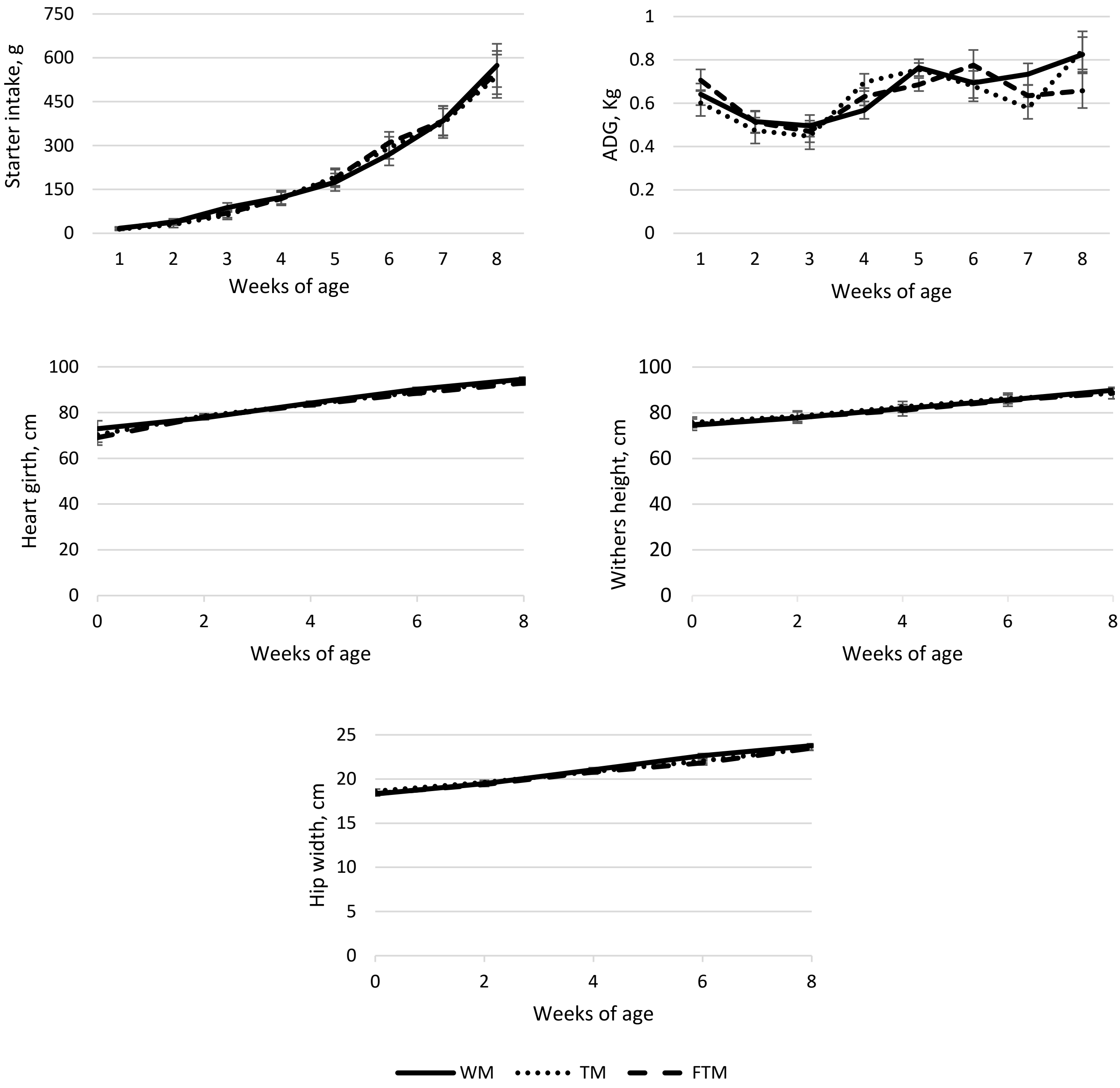

Figure S2. Starter intake, average daily gain, and body measurements of calves fed whole milk, transition milk or formulated transition milk for 3 days after colostrum feeding. P-value for treatment or treatment and age interaction effect are all > 0.05. P-value for age effect are all <0.0001.

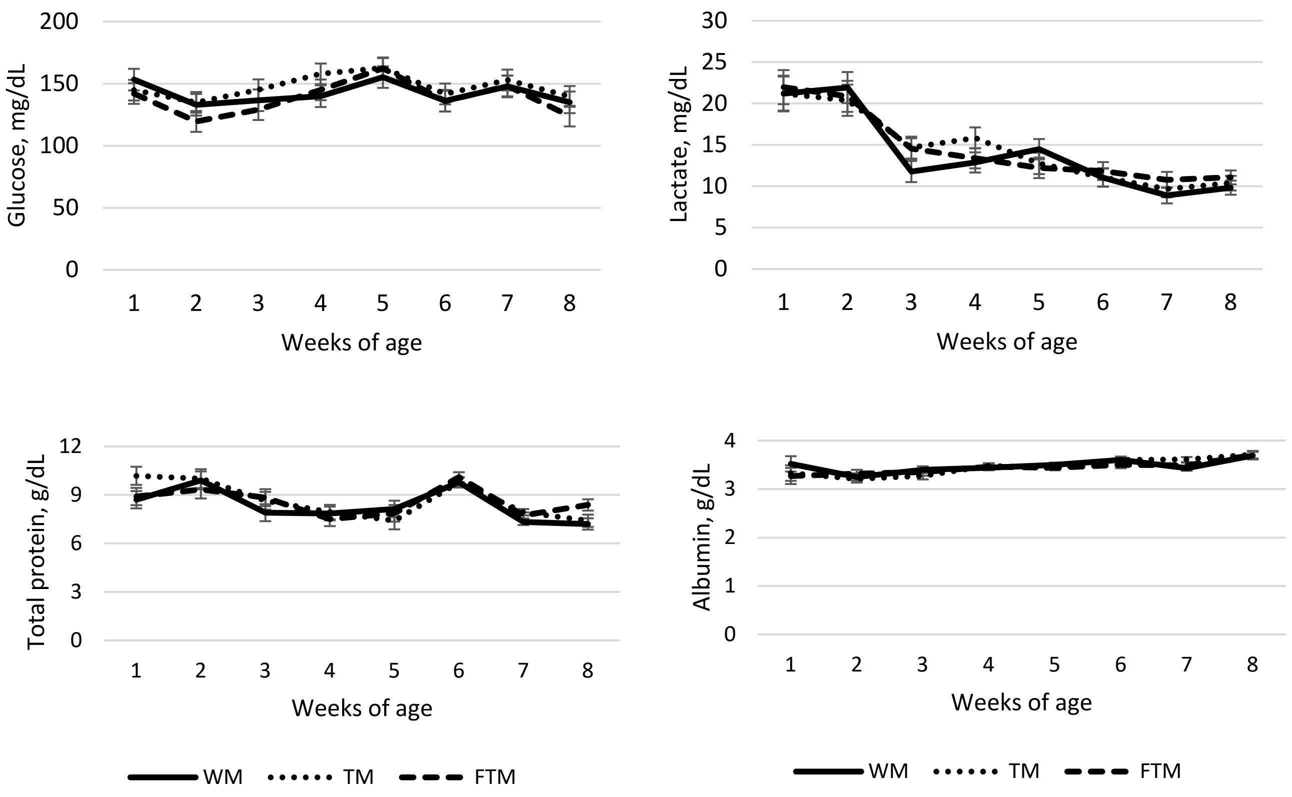

Figure S3. Preweaning blood parameters of calves fed whole milk, transition milk or formulated transition milk for 3 days after colostrum feeding. P-value for treatment or treatment and age interaction effect are all > 0.05. P-value for age effect are all <0.0001.
